# Photoreactive UV-Crosslinkable Acrylic Pressure-Sensitive Adhesives (PSA) Containing Multifunctional Photoinitiators

**DOI:** 10.3390/polym13244413

**Published:** 2021-12-16

**Authors:** Marcin Bartkowiak, Zbigniew Czech, Hyun-Joong Kim, Gyu-Seong Shim, Małgorzata Nowak, Adrian Krzysztof Antosik

**Affiliations:** 1 Department of Chemical Organic Technology and Polymeric Materials, Faculty of Chemical Technology and Engineering, West Pomeranian University of Technology, Szczecin, Pułaskiego 10, 70-322 Szczecin, Poland; psa_czech@wp.pl (Z.C.); nowak.malgorzata@zut.edu.pl (M.N.); Adrian.Antosik@zut.edu.pl (A.K.A.); 2 Laboratory of Adhesion and Bio-Composites, Program in Environmental Materials Science, College of Agriculture and Life Sciences, Seoul National University, Seoul 08826, Korea; hjokim@snu.ac.kr (H.-J.K.); sks6567@snu.ac.kr (G.-S.S.); 3 Research Institute of Agriculture and Life Sciences, College of Agriculture and Life Sciences, Seoul National University, Seoul 08826, Korea

**Keywords:** pressure-sensitive adhesives, multifunctional photoinitiators, UV-crosslinking

## Abstract

The use of ultraviolet radiation (UV) technology for the crosslinking of acrylic pressure-sensitive adhesives (PSA) is the one of various crosslinking methods, being the alternative to the conventional crosslinking process of solvent-based acrylic systems. It also requires a photoinitiator to absorb the impinging UV and induce photocrosslinking. As previously mentioned, a photoinitiator is one of the important and necessary components in UV-inducted crosslinking of acrylic pressure-sensitive adhesives. The activity of multifunctional conventional saturated photoinitiators of type I and type II, especially benzophenone-based in the photoreactive UV-crosslinkable acrylic PSA was described. The effect of the multifunctional type-II photoinitiators on the acrylic PSA, such as tack, peel adhesion and shear strength were summarized.

## 1. Introduction

The market for UV technology has been growing in the last years. A significant reason for this technology growth is its unique process characteristics, which allow UV-coating to be applied on virtually any substrates. UV technologies have also found their place in the production of acrylic PSAs. UV-crosslinked acrylic pressure-sensitive adhesives are made by UV-irradiation of the layer of base adhesive containing crosslinkable compounds, coated onto the carrier film [1].

**Figure 1:**
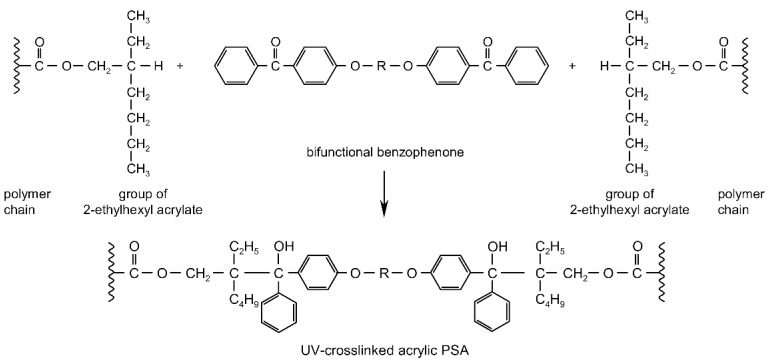
UV-initiated crosslinking reaction of PSA with multifunctional benzophenones (R = organic group).

UV-crosslinking technique represents a major advance in the development of the adhesive and self-adhesive coating industries [2,3,4,5,6,7,8,9,10]. The photoinitiator plays a key role in UV-crosslinkable systems by generating the reactive species, free radicals, which will initiate the crosslinking of photoreactive adhesives. Through its concentration controls directly both the crosslinking rate and the penetration of the UV radiation, and therefore the cure depth [11,12,13,14,15,16]. The efficiency of radical photoinitiators is dependent on a strong absorbance of the UV-radiation emitted by the UV-lamp, a short lifetime of the excited states to avoid quenching by atmospheric oxygen, a fast photolysis and bleaching, which generate the free radicals, a high reactivity of the free radicals evolved toward the monomer function, a good solubility of the photoinitiator in the formulation and the formation of non-colored and odorless photoproducts [15,17,18,19,20,21,22]. In the case of saturated acrylic pressure-sensitive adhesives, the photogeneration of initiator radicals by α-cleavage photoinitiators (Ph_I_) or H-abstraction photoinitiators (Ph_II_) is followed by reaction with the acrylic chain to produce a new radical that reacts with a neighboring acrylic chain [23]. This group of substances consists of saturated photoinitiators, which contain at least two photoreactive structures in the molecule and form crosslinkage with the pressure-sensitive adhesives by UV radiation [24]. It is possible to obtain so-called migration-free photoinitiators by specific constructions e.g., from multifunctional benzophenones. The UV-crosslinking mechanism of acrylic PSAs containing photoreactive multifunctional benzophenone derivatives has been thoroughly investigated and is presented schematically (Figure 1).

During UV exposure of the intermolecular benzophenone derivatives, H-abstractor structures are excited and react with the neighboring C–H positions of the polymer sidechains. UV-crosslinkable acrylic pressure-sensitive adhesives possess excellent oxidation resistance that allows working without inert gas atmosphere.

The target of this work was to evaluate the activity of novel multifunctional photoinitiators of two types (α-cleavage and H-abstractor) on the properties of crosslinked acrylic PSAs. Although the use of similar photoinitiators is described in the literature, it must be remembered that for products with specific performance characteristics, both the use of an appropriate photoinitiator and the composition of the base adhesive that undergoes crosslinking are important. Here, there are a huge number of possible combinations of acrylate adhesive composition and selected photoinitiators that can lead to a crosslinked pressure-sensitive adhesive of the desired strength. A new and original element of the presented research is the use of photoinitiators never described before. Furthermore, the composition of the base adhesive undergoing crosslinking was significantly different from that previously reported in the literature. In addition to 2-EHA and MA, it also contained a high proportion of BA, with typical AA content not exceeding 5 wt.%. Due to the growing demand for new efficient cross-linking photoinitiators in various branches of adhesives and coatings production, it is expedient to study the performance of new cross-linking systems, especially for the pressure-sensitive adhesive industry.

## 2. Materials and Methods

Various experiments were carried out to study the influence of different multifunctional photoinitiators type I (α-cleavage) and type II (H-abstractors) on the main performance of solvent-based acrylic pressure-sensitive adhesives (PSA), such as tack, peel adhesion (adhesion) and shear strength (cohesion). The base weight of the adhesive layer covering the polyester foil was 60 g/m^2^.

The influence of the crosslinking agents or crosslinking methods is usually determined in relation to the reaction time and to the concentration versus adhesion properties, considering the following properties: tack, adhesion, and cohesion. These mentioned properties were determined by standard A.F.E.R.A. (Association des Fabricants Europeens de Rubans Auto-Adhesifs) procedures. Exact details can be found in AFERA 4015 (tack), AFERA 4001 (peel adhesion) and AFERA 4012 (shear strength). Administrative address: 60, rue Auber-94408, Vitry Sur Seine Cedex, France.

The tests were carried out using tensile testing machine Zwick/Roell Z-25 (ZwickRoell GmbH & Co. KG, Ulm, Germany) and our own constructed machine for evaluation of shear strength due to AFERA 4012 standard (based on a programmable laboratory dryer with sample panels, appropriate sensors, computer and recording software).

The tack of pressure sensitive adhesives is a property of an adhesive that occurs upon brief contact of a test material with a standard surface and represents the force required to de-bond a sample from that surface. The tack test method due to AFERA 4015 is simple and requires common tensile testing machine. The strip of tested material (carrier tape coated with PSA) with dimensions of 1 × 7 inches is folded to clamp its ends in the jaws of testing machine. Then the loop formed is contacted with horizontal clean steel plate (without applying additional pressing force), and the force required to separate the loop from the plate is measured.

The peel adhesion is the force required to separate a PSA coated strip of carrier tape from a standard test plate with constant rate speed of removal and at a specific angle of removal. The AFERA 4001 peel adhesion test is carried out with strip of tested material (carrier tape coated with PSA) with dimensions of 1 × 5 inches, bonded firmly with additional pressing force (2 kg rubber roller) to the clean steel plate. The plate and the free end of the strip are clamped in the jaws of testing machine to provide 180° angle of removal. Then the force required to peel off the tape from the plate is measured.

The shear strength is the measure of the cohesiveness (internal strength) of an adhesive. According to the AFERA 4012 standard, the method consists in measuring the force necessary to shear the sample of PSA layer, acting in a direction parallel to the surface of the contact. The measurement is carried out in temp. 20 °C (5–90 N of the load) and 70 °C (5–40 N of the load). Adhesive-coated strip is applied from one end to clean steel panel (1 × 1 inch of contact area), and free end of the strip is loaded with a force of different hanging weight for given time (4 h).

The amount of solid materials was determined by weight after drying, the residual of monomers were measured with gas chromatograph Unicam 610, J&W DB-1 column, FID detector and integrator Unicam 4815.

The molecular weight studies were performed with a liquid chromatograph LaChrom system: RI Detector L-7490 and LaChrom UV Detector L-7400 from Merck Hitachi, equipped with a PLgel 106 Å column from Hewlett Packard.

The evaluated photoreactive pressure-sensitive adhesives were crosslinked using ultraviolet light lamp Aktiprint-mini 18-2 from Technigraf Company (Grävenwiesbach-Hundstadt, Hessen, Germany) and the UV-exposure can be measured using an integrating radiometer DynachemTM Model 500, available from Dynachem Corporation (Caronno Varesino, Italy).

### 2.1. Basic Acrylic PSA

The following experiments were conducted using standard solvent-based acrylic PSA synthesized from 40 wt.% of 2-ethylhexyl acrylate, 30 wt.% of butyl acrylate, 25 wt.% of methyl acrylate and 5 wt.% of acrylic acid in the organic solvent ethyl acetate at the boiling point temperature about 77 °C at presence of 0.1 wt.% 2,2′-azo-bis-diisobutyronitrile (AIBN), according to monomers mixture concentration, used as thermal initiator to start radical polymerization. All starting materials such as acrylate monomers, solvent and AIBN were available from BASF (Germany). All investigated multifunctional photoinitiators type I and type II were technical grade and were synthesized at UTP University of Science and Technology, Faculty of Chemical Technology and Engineering, Department of Organic Chemistry.

The final synthesized solvent-based basic acrylic PSA was characterized by the following significant properties:

Amount of solid materials 50 wt.%
Viscosity 10.3 Pa·s
Concentration of residual monomers <0.2 wt.%
Weight average molecular weight MW 621,000 Da
Number average molecular weight Mn 214,000 Da
Polydispersity Pd = MW/Mn 2.90

### 2.2. Investigated Photoinitiators Type I (α-Cleavage) and Type II (H-Abstractors)

Examples of the tested type I and type II multifunctional photoinitiators are presented in Table 1, which were tested as photoreactive crosslinking agents for solvent-based acrylic PSA without tertiary amine co-initiators. This class forms at least two photoreactive structures in the polymer molecule and forms crosslinkage with the polymer chains via ultraviolet radiation. The most typical direction, however, is in the development of multifunctional benzophenones.

## 3. Results and Discussion

**Figure 2:**
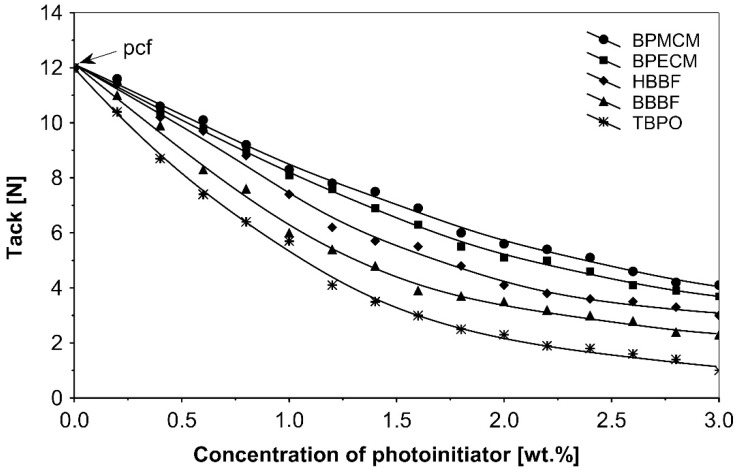
Influence of multifunctional photoinitiators on the tack of a UV-crosslinked acrylic PSA.

**Figure 3:**
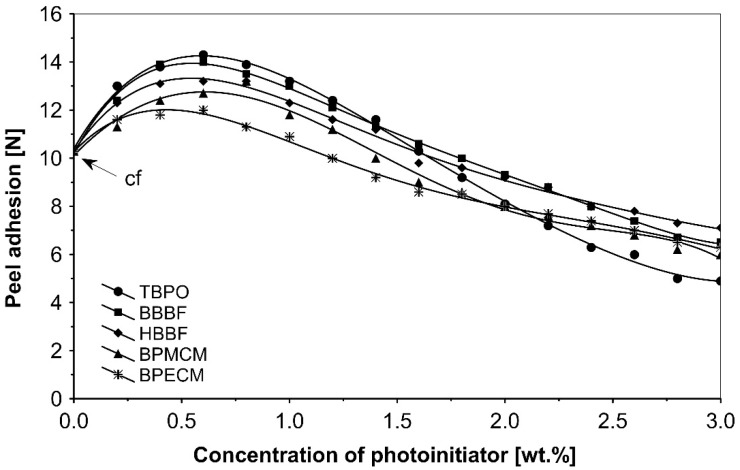
Influence of multifunctional photoinitiators on the peel adhesion of a UV-crosslinked acrylic PSA.

**Figure 4:**
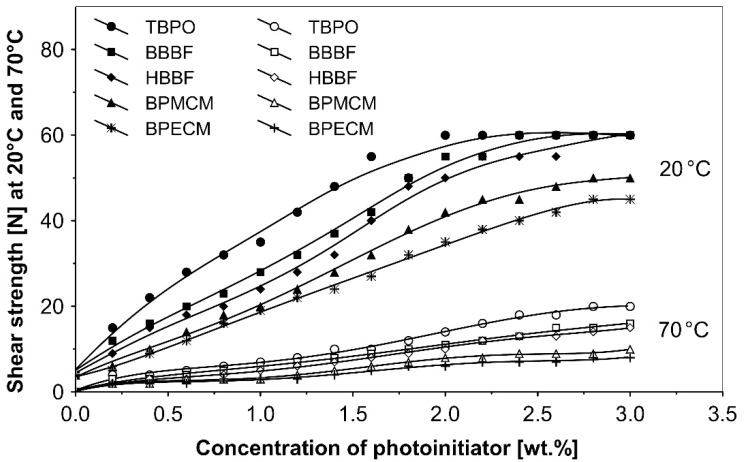
Influence of multifunctional photoinitiators on the shear strength of a UV-crosslinked acrylic PSA.

The UV-crosslinking effect of the examined multifunctional photoinitiators of type I and type II used with a concentration between 0.2 to 3.0 wt.%) in basic acrylic PSAs, on tack, peel adhesion and shear strength, using 100 mJ/cm^2^ UV dose after 3 min UV exposure, is presented in Figure 2, Figure 3 and Figure 4.

UV-crosslinked acrylic pressure-sensitive adhesives containing multifunctional saturated photoinitiators shows different tack (Figure 2) and peel adhesion (Figure 3) profiles depending on the concentration and the kind of photoinitiator. Figure 2 and Figure 3 give typical examples, decrease of tack with increase of photoinitiator amount and a maximum of peel adhesion for small amount of photoinitiator ranging between 0.4 and 0.8 wt.%. The multifunctional H-abstractors are efficient photoinitiators, although less compared to multifunctional photocleavable derivatives. The best peel adhesion was activated by the application of the trifunctional H-abstractor tris-benzophenyloxy phosphineoxide (TBPO) and the bifunctional butanediol-1,4-bis-benzophenoxy formiate (BBBF). The abbreviation *pcf* means partially cohesive failure, and *cf* means cohesive failure.

The resistance to creep in a shear strength test (Figure 4) increases with the elevation of the saturated multifunctional photoinitiator′s concentration. The best shear strength values of UV-crosslinked acrylic PSAs were observed by using multifunctional hydrogen atom abstractors. The best shear strength at 20 °C and at 70 °C occurred with the H-abstractors like tris-benzophenyloxy phosphineoxide (TBPO) and butanediol-1,4-bis-benzophenoxy formiate (BBBF).

The best balance between the main performance of UV-crosslinked acrylic PSAs, reflected the combination of tack, peel adhesion and shear strength, in the investigated adhesive layer can be achieved with 0.4 to 1.2 wt.% of tris-benzophenyloxy phosphineoxide (TBPO).

**Figure 5:**
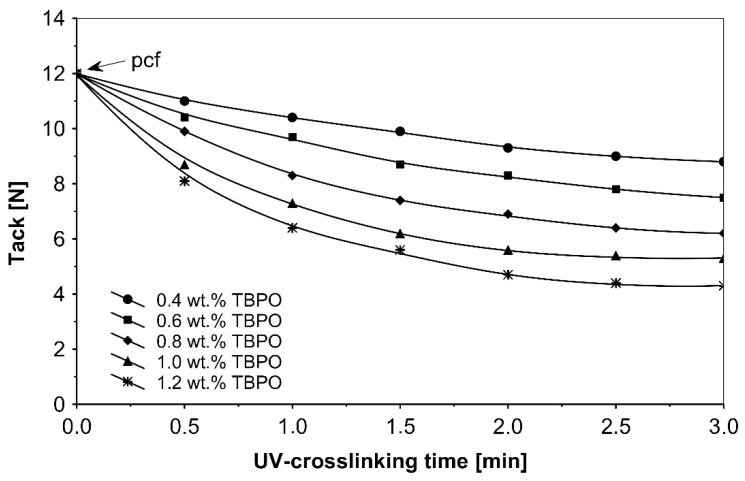
Tack versus UV-crosslinking time for the use of tris-benzophenyloxy phosphineoxide (TBPO).

**Figure 6:**
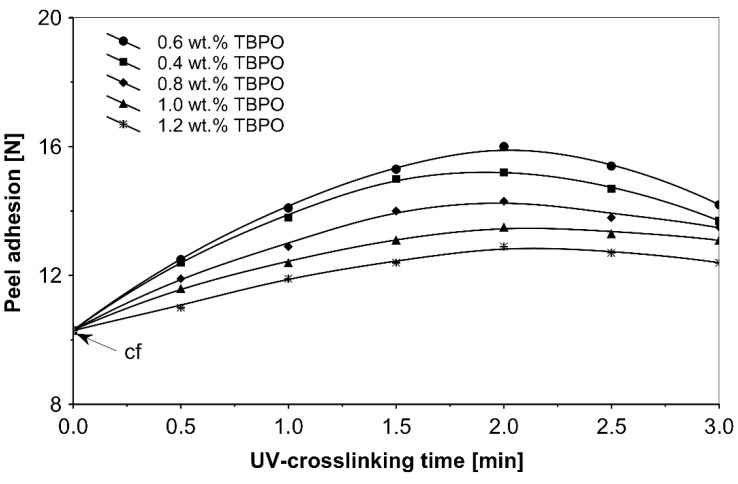
Peel adhesion versus UV-crosslinking time for the use of tris-benzophenyloxy phosphineoxide (TBPO).

**Figure 7:**
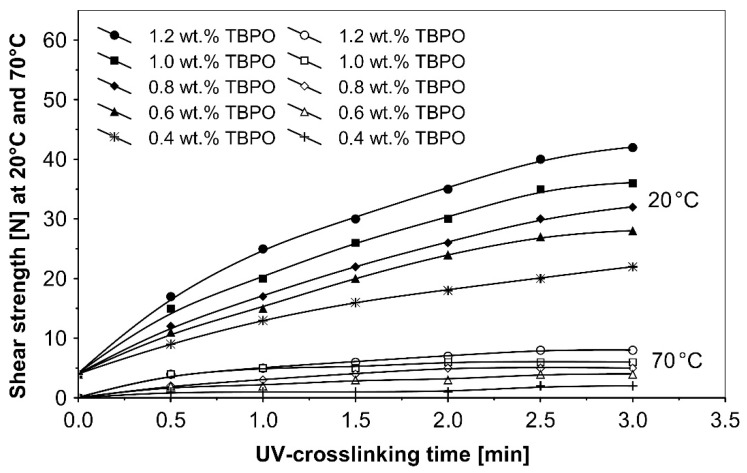
Shear strength versus UV-crosslinking time for the use of tris-benzophenyloxy phosphineoxide (TBPO).

**Figure 8:**
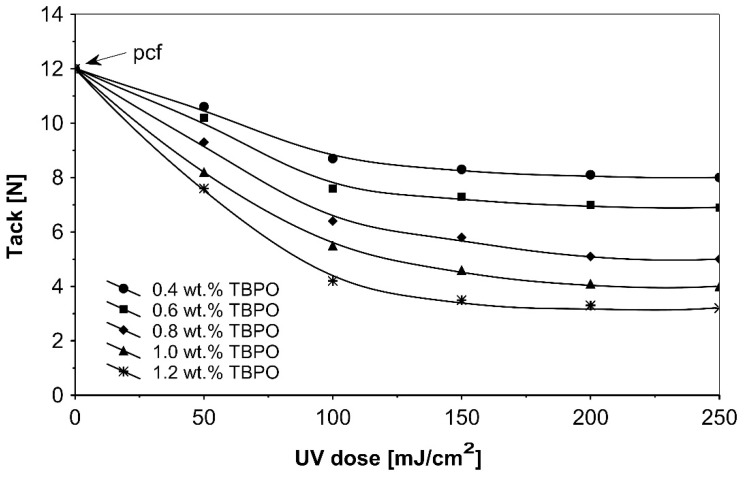
Tack versus UV dose for the use of tris-benzophenyloxy phosphineoxide (TBPO).

**Figure 9:**
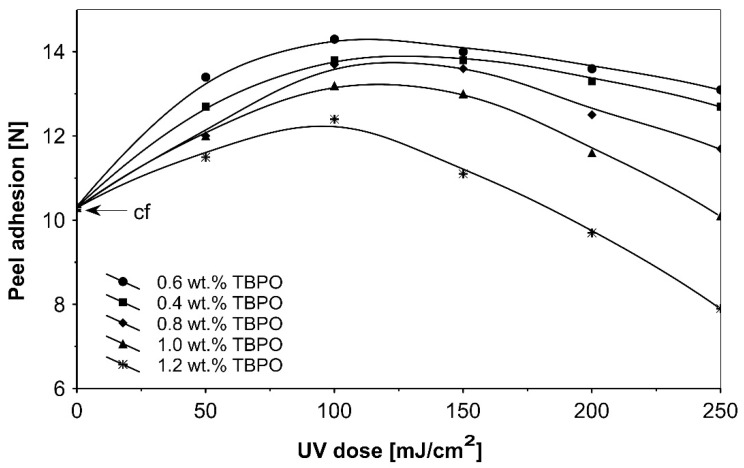
Peel adhesion versus UV dose for the use of tris-benzophenyloxy phosphineoxide (TBPO).

**Figure 10:**
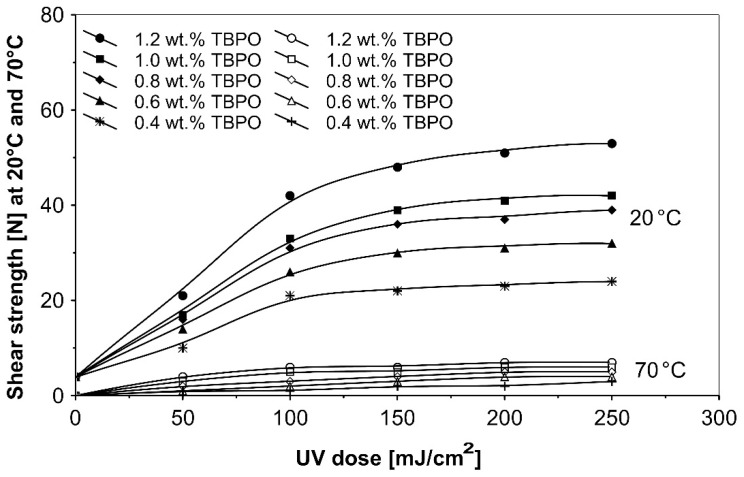
Shear strength versus UV dose for the use of tris-benzophenyloxy phosphineoxide (TBPO).

Further trials, as to UV-crosslinkable acrylic PSAs containing the best multifunctional type II photoinitiator tris-benzophenyloxy phosphineoxide (TBPO) in selected amounts between 0.4 to 1.2 wt.%, were conducted with different UV-crosslinking windows and by using UV doses up to 250 mJ/cm^2^. The experimental results of these investigations are presented in Figure 5, Figure 6, Figure 7, Figure 8, Figure 9 and Figure 10.

As in the previous trials, the tack of UV-crosslinked acrylic pressure-sensitive adhesives will decrease with increasing of the concentration multifunctional H-abstractor TBPO and with increasing UV-crosslinking time. The highest tack values were noticeable for un-crosslinked adhesives with partially cohesive failure.

As can be seen in Figure 6, it is very difficult to predict the maximum peel adhesion range for UV-crosslinked adhesives containing 0.6 wt.% and 0.4 wt.% tris-benzophenyloxy phosphineoxide (TBPO) at a UV-crosslinking time of 2 min, because the physical properties of the PSA, such as peel adhesion, are also affected by other factors, like chemical architecture of the polymer side chain and an amount of free radicals from multifunctional hydrogen atom abstractors. For higher concentrations of TBPO, and with a little bit an increase of UV-crosslinking time above 2 min, the peel adhesion suffers a slight decrease. When high peel adhesion is attained at about 2 min UV exposure, when high peel adhesion is attained at about 2 min UV exposure, the shear strength at room temperature and at 70 °C (Figure 7) reaches a satisfactory value. The cohesion of UV-crosslinked acrylic PSAs increases significantly with the UV exposure time.

Figure 8 presents the results for the tack, and Figure 9 presents the results for the peel adhesion of UV-crosslinked acrylic PSAs in relation to UV dose applied. It is well known that higher ultraviolet doses negatively influence the tack and favorably change the peel adhesion, in an area between 50 and 150 mJ/cm^2^. Moreover, the shear strength (Figure 10) increases significantly. Very close adhesive values are obtained for peel adhesion of acrylic PSAs containing 0.4 to 0.8 wt.% of tris-benzophenyloxy phosphineoxide (TBPO). Such results are difficult to be interpreted.

Figure 10 shows the effect on the shear strength of pressure-sensitive adhesives containing various amounts of the three-functional type II photoinitiator tris-benzophenyloxy phosphineoxide (TBPO), at different radiation doses from a UV lamp.

As can be seen in Figure 10, from the shear strength results at 20 °C and 70 °C for variable UV doses, the most efficient curing of acrylic adhesive will be observed for PSAs having 1.2 wt.% TBPO and crosslinked with 250 mJ/cm^2^ ultraviolet exposure. The measured shear strength values at 70 °C were relatively low.

The increase of the shear strength can be explained by the increase of the internal structure stability of adhesive layer. The main influence on this stability is the crosslinking density of the polymer [10,15]. Relation between shear strength of any PSA and concentration of crosslinking agent and/or UV dose is generally typical for every type of crosslinked PSAs [25]. It is similar with the general dependence that with increasing crosslinking density and shear strength, peel adhesion and tack decrease. These dependencies can be observed in the results of many researchers, regardless of the adhesive components and crosslinking agents used [26].

Comparing the presented results of research on completely new photoinitiators with the current research on crosslinking of relatively similar acrylate PSAs [27,28,29], a significant increase in all three performance parameters can be noticed. However, it should be noticed that it is not always possible to directly compare the results, especially since the properties of the adhesive are influenced not only by the method and effect of crosslinking, but first by the composition of the base adhesive [30].

Summarizing the results of this chapter it can be concluded that the search for new multifunctional photoinitiators of type I and type II is worthwhile. The results of performance of adhesives crosslinked with them are very promising, especially since a better balance of the properties (tack-adhesion-cohesion) is achieved as in the case of previously described photoinitiators [31].

Multifunctional H-abstractors, based on benzophenone derivatives, brought a better performance (like their photocleavable photoinitiator equivalents) because they had the potential to produce two or more different radical initiating species. UV-crosslinkable acrylic pressure-sensitive adhesives containing one- or multifunctional hydrogen atom abstractors can pave the way for manufacturing pressure-sensitive adhesives with optimum performance properties.

## 4. Conclusions

The following conclusions are derived from the experiments carried out with multifunctional intermolecular hydrogen atom abstraction type II photoinitiators:

- There is a clear dependence of shear strength of UV-crosslinked acrylic PSA on photoinitiator concentration.
- The increase of the photoinitiator concentration reduces the tack of UV-crosslinked acrylic adhesives, the peel adhesion reaches the maximum at about 0.6 wt.% of type II photoinitiator of tris-benzophenyloxy phosphineoxide (TBPO).
- From the examined multifunctional hydrogen atom abstractors, the best performances were noticed in the case of the type II photoinitiator of tris-benzophenyloxy phosphineoxide (TBPO). Comparative studies between the UV-crosslinked acrylic PSA containing the selected photoinitiators type II and TBPO showed a little superiority of the UV-crosslinkable basic self-adhesive containing tris-benzophenyloxy phosphineoxide.
- The UV-crosslinkable acrylic PSA containing TBPO can be used for manufacturing of self-adhesive materials in form of mounting tapes, masking tapes or wide range of sign and marking films.

## Figures and Tables

**Table 1:** Multifunctional photoinitiators of type I and type II used for UV-crosslinking of acrylic PSAs.

Photoinitiator	Chemical Formula	Chemical Name
BPMCM	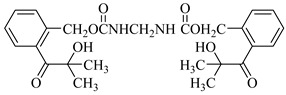	Bis-[2-(2-hydroxy-2-methyl-propione-1-one)phenylmethylene carbamyl] methylene
BPECM	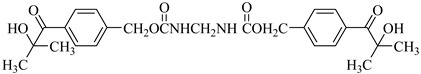	Bis-[4-(2-hydroxy-2-methyl-propione-1-one)phenylethylenecarbamyl] methylene
BBBF	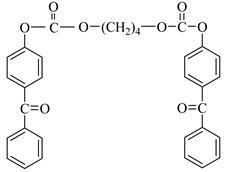	Butanediol-1,4-bis-benzophenoxyformiate
HBBF	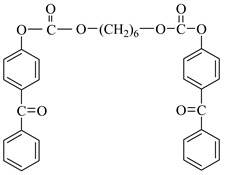	Hexanediol-1,6-bis-benzophenoxyformiate
TBPO	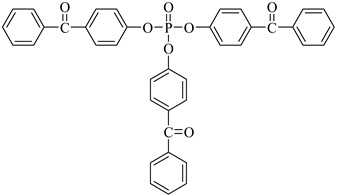	Tris-benzophenyloxy phosphineoxide

## Data Availability

The data presented in this cannot be shared at this time as this data forms part of an ongoing study.
